# Resident duty hours: past, present, and future

**DOI:** 10.1186/1472-6920-14-S1-S1

**Published:** 2014-12-11

**Authors:** Kevin R Imrie, Jason R Frank, Christopher S Parshuram

**Affiliations:** 1 Sunnybrook Health Sciences Centre, Toronto, Ontario, Canada; 2 The University of Toronto, Toronto, Ontario, Canada; 3 The Royal College of Physicians and Surgeons of Canada, Ottawa, Ontario, Canada; 4 The Department of Emergency Medicine, University of Ottawa, Ottawa, Ontario, Canada; 5 The Hospital for Sick Children, Toronto, Ontario, Canada

## Background

Postgraduate medical education is founded on a tradition of service and education and is grounded in the realities of the twenty-first century. The very nature of the postgraduate enterprise is evolving in front of us all, and this is having a wide-ranging impact in a variety of areas. At stake is both the delivery of quality patient care and the preparation of the next generation of competent physicians. The engagement of the medical profession is an essential part of this reformation.

One of the fundamental questions facing contemporary medicine is how best to organize the work hours of physicians who are undergoing clinical training. The postgraduate medical education of the physicians we refer to as “residents” is recognized by those within and outside medicine as an enterprise dedicated to preparing new physicians to enter independent practice. It is a time of great commitment for all involved – to learning, to patient care, and to the professional development of future leaders of health care teams. It is also a period that can be experienced through a number of perspectives: that of the residents and the patients they care for, the faculty who teach them, the training programs that support their progress, the certifying bodies that ultimately endorse them for entry into practice, and the administrators in the teaching hospitals where residents learn to balance their dual roles as learners and service providers on a health care team. These perspectives differ and diverge, and they also, ultimately, lead to the inherent tensions within residency in the modern era: education versus service, supervision versus autonomy, and quantity versus quality of care, among many others.

Throughout much of the world, the hours that residents work have gradually decreased since the 1980s. This change has accelerated within the past five years with recent legislative and regulatory changes in Europe, the United States, Canada, and other jurisdictions. The question of how long residents should work has been the subject of heated discussion within the profession and among the broader public for almost three decades.

The impetus for this special issue of *BMC Medical Education* arose from symposia on the topic of resident duty hours held at the annual International Conference on Residency Education in 2010 and 2011. These symposia, entitled respectively “Duty hours across borders” and “Solutions across borders,” took place as the most recent round of duty hour reforms were announced and implemented in the United States, the United Kingdom, and Canada, and both symposia included presentations by many of the authors of papers included in this issue. Importantly, these sessions provided an international forum to explore the challenges that can arise as a result of – and potential solutions to – changing duty hours, a dialogue we have capitalized on with this special issue.

## A brief history of duty hour regulations

To accurately interpret the published data, it is essential to understand the historical context and the specifics of prior duty hour regulations.

Early in the twentieth century, residents in North America actually resided in hospitals, providing after-hour “on-call” medical services as part of their training [1]. Until the initial reforms to duty hours a century later, residents frequently worked more than 90 hours per week, with each week comprised of 36-hour shifts separated by 12 hours or less of rest [2].

The impetus to restrict duty hours varied from jurisdiction to jurisdiction. An initial catalyst for change in North America was the death of a college student, Libby Zion, in 1984 [3]. Concerns over resident duty hours, as well as the lack of supervision evident in this case, led to the formation of the Bell Commission in the state of New York. The Commission recommended improved supervision of residents and reforms to duty hours. These recommendations led, in 1989, to a New York State Department of Health Code, also known as the Libby Zion law, which limited residents’ hours of work [4]. In 2003, the Accreditation Council for Graduate Medical Education (ACGME) implemented standards that limited the hours of work of residents across the United States to 80 per week, with no more than 30 consecutive hours of work [5]. In 2011, in response to both a report published by the Institute of Medicine and evidence that the 2003 duty hour restrictions had done little to reduce resident fatigue or improve patient safety, the ACGME implemented more restrictive standards for the United States [1,6].

Also in 2011, in Canada, the province of Quebec ruled that shifts of 24 hours or more contravened both the provincial and national charters of rights and freedoms. As a result, duty hour schedules in the province were limited to no more than 16 continuous hours.

In the European Union (EU), the movement to limit resident duty hours followed a somewhat different path. The European Working Time Directive (EWTD) was implemented in 1998, establishing the right of EU workers not to be required to work excessively long hours. This affected a number of professions, including those in the health sector. In the United Kingdom, doctors in training were exempted from these restrictions until 2004. Beginning in that year, hours for doctors in training were gradually reduced, finally reaching in 2009 a maximum of 48 per week, with no more than 13 consecutive hours of work [7].

Resident work hours have also decreased in Australia and New Zealand over the past 20 years. As in Europe, the major driver of this change has been resident well-being rather than patient safety.

A detailed survey of the current state of duty hour limits across these jurisdictions can be found in this issue in Sir John Temple’s paper “Resident duty hours around the globe: where are we now?” [8]

## In this issue

This issue examines the impact of changing resident duty hours through a number of lenses. Changes to the hours that residents work can be expected to have different effects on resident satisfaction and well-being, medical education, teaching faculty, patient safety, and the structure of health care delivery in teaching hospitals. These effects may vary by jurisdiction, reflecting differences in the structures of both health care delivery and medical education systems.

The challenges and solutions may also differ between specialties, as the nature of practice and learning differs. As we assembled this special issue of *BMC Medical Education*, we felt it essential that these perspectives be reflected and explored. An international panel of expert authors provides historical and current perspectives across different domains of medical education and health service delivery. The crucial voice of residents is well represented from a range of jurisdictions. The result is a rich array of distinct scholarly perspectives on this important topic.

The first set of papers in this issue address the intersection of duty hours with patient safety [9], resident well-being [10], the health system [11], and teaching faculty [12]. In the next eight papers, we present perspectives from different jurisdictions around the globe, including a detailed exploration of the challenges and current conditions in Sweden [13], the United States [14,15], Canada [16,17], the United Kingdom [18], and Australia [19], with articles written from the perspectives of both residents and faculty. In this section, we also include papers that address the unique considerations for procedural disciplines [20], as well as the impact of duty hour changes on professionalism [21].

This special issue culminates with a discourse on the important issue of finding our way forward. Three solutions-focused papers explore strategies for adapting to changing resident duty hours. These articles examine how to improve continuity of care [22], a new model for the provision of after-hours care [23], and novel work schedules for providing in-patient care in the era of restricted duty hours [24].

As the editors of this special edition, we are pleased to be sharing the fruits of our international journey of residency education. These perspectives from around the globe articulate commonalities and differences from which we can learn and develop new ideas. We thank the authors for thoughtful contributions that describe their realities and solutions, and we hope that you will find the result thought-provoking, insightful, and valuable.

Supplement Co-Editors:

Dr. Kevin Imrie, MD, FRCPC

Physician-in-Chief, Sunnybrook Health Sciences Centre

Vice-Chair, Education, Department of Medicine, University of Toronto Professor of Medicine, University of Toronto

Dr. Christopher Parshuram, MB, ChB, D.Phil.

Physician, Critical Care Program

Senior Scientist, Child Health Evaluative Sciences, The Research Institute, Hospital for Sick Children

Associate Professor Paediatrics, Critical Care, Health Policy, Management & Evaluation Faculty, Centre for Quality Improvement and Patient Safety, University of Toronto

Dr. Jason Frank, MD, FRCPC

Director, Specialty Education, Strategy, and Standards, Office of Specialty Education, The Royal College of Physicians and Surgeons of Canada

## Authors’ contributions

All authors contributed to the concept, drafts, and revisions of this manuscript. All approved the final version.

## Competing interests

The supplement co-editors and authors declare that they have no competing interests.

## Declarations

Resources and secretariat support for this project was provided by the Royal College.

This article has been published as part of *BMC Medical Education* Volume 14 Supplement 1, 2014: Resident duty hours across borders: an international perspective. The full contents of the supplement are available online at http://www.biomedcentral.com/bmcmededuc/supplements/14/S1.

Publication of this supplement was supported and funded by the Royal College of Physicians and Surgeons of Canada. The funding agency played no role in the design, in the collection, analysis and interpretation of data; in the writing of the manuscripts; and in the decision to submit the manuscript for publication. The articles have been through the journal's standard peer review process for supplements. The Supplement Editors declare that they have no competing interests.
